# Match Injury Incidence for a New Zealand Amateur Domestic Female Soccer Team over Two Consecutive Seasons

**DOI:** 10.3390/sports12080216

**Published:** 2024-08-09

**Authors:** Doug A. King, Patria A. Hume, Trevor N. Clark

**Affiliations:** 1 Auckland Bioengineering Institute, The University of Auckland, Auckland 1023, New Zealand; phume@aut.ac.nz (P.A.H.); trev.waves@gmail.com (T.N.C.); 2 Sports Performance Research Institute New Zealand (SPRINZ), Faculty of Health and Environmental Science, Auckland University of Technology, Auckland 1142, New Zealand; 3 School of Science and Technology, University of New England, Armidale 2351, Australia; 4 Wolfson Research Institute for Health and Wellbeing, Department of Sport and Exercise Sciences, Durham University, Durham DH1 3LE, UK; 5 Technology and Policy Lab, Law School, The University of Western Australia, Perth 6009, Australia

**Keywords:** football, soccer, female, amateur, injury incidence, injury probability

## Abstract

Objective: To determine the match injury incidence for a New Zealand amateur domestic female soccer team over two consecutive seasons. Methods: A descriptive, epidemiological observational study was conducted to determine match injury incidence for 49 players over two domestic seasons. Match exposure and injury burden were calculated. Results: A total of 84 match-related injuries resulted in a match injury incidence of 145.5 (95% confidence interval (CI): 117.4 to 180.1) per 1000 match h. Attackers had a higher incidence of injuries for total (200.0 per 1000 match h) and missed matches (152.4 per 1000 match h). The lower limbs had the highest injury incidence (105.6 per 1000 match h), with ankle injury being the most reported (43.3 per 1000 match h) lower limb injury. Over three quarters (75.3%) of the injuries recorded were missed match injuries. Sprains/strains were the most recorded total (86.6 per 1000 match h) injury type. Fractures were recorded as having the highest mean injury burden (68.7 ± 70.4 days). Discussion: Historically, there was a paucity of injury burden data for female football; however, the data presented within this study can be utilised to support the identification of injury patterns and areas to be included within injury reduction programmes.

## 1. Introduction

There has been an increase in the professionalism and participation numbers of female team sport athletes [1]. Specific to football (hereinafter termed soccer), the Federation Internationale de Football Association (FIFA) reported a 24% growth in women and girls playing organised football between 2019 and 2023 [2]. In Australia, there was a 21% increase in female participation between 2020 and 2021 [2], whilst there was a 25% increase in female players within New Zealand between 2022 and 2023. However, despite the increase in participation, there is a scarcity of research exploring injuries in this cohort [3], and a recent call to action [1] highlighted the need to increase the evidence base concerning women’s injuries in sport to help inform practice. Data obtained from studies involving males cannot be generalized to females [4], as they exhibit differences in fatigability, responses, and performance [5]. As such, there is a relative lack of published literature surrounding injury surveillance within women’s soccer [6], with Northern hemisphere data indicating that injuries most frequently occur to the knee, ankle, and thigh (i.e., lower limb) [7].

In a recent systematic review [8] reporting on the epidemiology of women’s soccer injuries, it was identified that there were no epidemiological studies identified at any level of participation in the Southern Hemisphere. Data on amateur female soccer player injuries in New Zealand is limited, where, to the authors’ knowledge, only one study [9] has ever been published. This study [9] undertook injury surveillance through phone contact with soccer players from two of the federations in New Zealand, using a player recall system for data collection. Consequently, there have still been no prospective observational cohort studies reporting on injuries within female soccer competitions in New Zealand. Such data are required to support the development and wellbeing of recreational to amateur female soccer players through informing targeted injury reduction programmes within domestic competitions. Therefore, the purpose of this study was to document and report on the incidence of match injuries of a club-based female amateur soccer team in a local competition over two consecutive years.

## 2. Methods

### 2.1. Protocol

A descriptive, epidemiological observational study was conducted to record the incidence of match injuries in an amateur female soccer team over two domestic seasons (2022 and 2023). The observation period spanned from January to September, with competition games taking place from April to August each season. The data were gathered in the New Zealand autumn and winter, which is similar to when soccer is played in Europe. The team being reported on participated in the Wellington female premier soccer competition, comprised of eight teams. Throughout the study, 49 players took part, with an average age of 23.0 ± 9.8 years. All participants were classified as amateurs since their primary income came from sources other than soccer and they did not receive any match payments. Before the competition season began, all players gave written consent to participate in the research, and all procedures received approval from the institutional ethics committee.

The injury definition utilised for this study was “any physical complaint which is caused by a transfer of energy which exceeds the ability of the body to maintain its structural and/or functional integrity that is sustained by a player during a soccer match, irrespective of the need for medical attention or time-loss from soccer activities” [10]. The methodological approach to this study is identical to previous studies [11,12,13,14,15,16] and enables comparisons to be made between the injuries in these sports in New Zealand. During the study, all match injuries were recorded to enable further analysis during total and time-loss analysis. All concussions were assessed with the concussion check protocol [13].

### 2.2. Statistical Analyses

All collected data were input into a Microsoft Excel spreadsheet and analyzed using the Statistical Package for Social Sciences for Windows (SPSS; V25.0.0). Match exposure was determined by considering 11 players exposed for 90 min. Positional group exposure was calculated based on the number of players in each group (i.e., defender, midfielder, attacker, and goalkeeper using a 4:4:2:1 allocation) being exposed for 90 min. Players’ ages were compared between 2022 and 2023 with a *t*-test, and the number of concussions recorded per year were compared with a one-sample chi-squared (*χ*^2^) test. Injury incidence rates (IIR) for match-related female soccer injuries were calculated utilising previously established calculations [17]. The injury burden was calculated as the time away from the match or team training participation following an injury, utilising time-loss calculation for the reporting of epidemiological data [12]. This was reported as the mean number of days lost with standard deviation (±SD). For interstudy comparisons, the injury burden was also calculated as the number of days lost per 1000 match h ((Σ days lost/Σ exposure h) × 1000) with 95% CI) [12]. Post hoc probabilities of female soccer match-related injuries occurring over a competition season were also determined utilising a Poisson distribution as previously described [15].

An independent *t*-test was employed to compare the mean difference in days lost. A one-sample chi-squared (*χ*^2^) test was utilised to assess whether the observed injury frequency significantly deviated from the expected injury frequency by competition year and for total injuries recorded. Risk ratios (RR) were used to compare injury rates per year, including total and missed match (MM) injuries [16]. The RR were assumed to be significant at *p* < 0.05. A two-sample *t*-test was used to assess differences in injury burden by competition year.

## 3. Results

Forty-nine players were enrolled in the study over the two years. Only 11 players participated across both seasons. The playing group was older in 2023 (25.1 ± 10.8 yr.) compared with the group in 2022 (20.4 ± 7.2 yr.; *t*_(28)_ = −1.7; *p* = 0.0488) (see Table 1). Over the study there were 84 match-related injuries recorded, resulting in a match injury incidence of 145.5 (95% CI: 117.4 to 180.1) per 1000 match h (see Table 1). There were more total (49 vs. 35; *χ*^2^_(1)_ = 1.6; *p* = 0.2054) and MM (39 vs. 27; *χ*^2^_(1)_ = 1.6; *p* = 0.2130) injuries recorded in 2022 when compared with 2023, but this was not significant.

Although attackers had a higher incidence of total (200.0 [95% CI: 130.4 to 306.7] per 1000 match h) and MM (152.4 [95% CI:93.4 to 248.7] per 1000 match h) injuries than goalies (Total: *χ*^2^_(1)_ = 2.3; *p* = 0.1272; MM *χ*^2^_(1)_ = 1.6; *p* = 0.2059), defenders (*χ*^2^_(1)_ = 0.9; *p* = 0.3314; MM *χ*^2^_(1)_ = 0.6; *p* = 0.4395), and midfielders (Total: *χ*^2^_(1)_ = 2.7; *p* = 0.0989; MM *χ*^2^_(1)_ = 1.3; *p* = 0.2513), this was not significant (see Table 2). Despite this, attackers were recorded as having a significantly higher injury burden for total (26.8 ± 42.3 days) and MM (34.3 ± 46.2 days) injuries than the goalies (Total: *χ*^2^_(1)_ = 7.7; *p* = 0.0056; MM: *χ*^2^_(1)_ = 7.0; *p* = 0.0080), defenders (Total: *χ*^2^_(1)_ = 199.8; *p* < 0.0001; MM *χ*^2^_(1)_ = 202.3; *p* < 0.0001), and midfielders (Total: *χ*^2^_(1)_ = 130.8; *p* < 0.0001; MM: *χ*^2^_(1)_ = 124.9; *p* < 0.0001). The defenders had the highest probability of an injury occurring for total (64.5%) and MM (75.4%) injuries.

There were significantly fewer match injuries in the first quarter for total (48.5 [95% CI: 23.1 to 101.7] per 1000 match h) injuries than the second (RR: 5.0 [95% CI: 2.2 to 11.2]; *p* < 0.0001), third (RR: 3.1 [95% CI:1.4 to 7.3]; *p* = 0.0053), and fourth (RR: 2.9 [95% CI:1.2 to 6.7]; *p* = 0.0124) match quarters (see Table 2). However, there were no observable differences in the incidence of injuries between match halves for total (*χ*^2^_(1)_ = 0.0; *p* = 1.0000) and MM (*χ*^2^_(1)_ = 0.5; *p* = 0.4602) injuries over the duration of the study. The 2nd quarter was recorded as having the highest probability of an injury occurring for total (95.8%) and MM (76.6%) injuries.

Twisting was the most common injury cause for total (46.8 [95% CI: 32.1 to 68.2] per 1000 match h) and MM (31.2 [95% CI: 19.6 to 49.5] per 1000 match h) injuries. Although contact with the playing surface (449 days) had the highest MM injury burden, overuse injuries resulted in the highest mean days lost for total (42.0 ± 72.2) and MM (82.0 ± 96.2) injuries. There was a higher probability that injuries that were a result of overuse would result in a MM (84.7%) than a non-missed match injury (79.2%).

Over three quarters (75.3%) of the injuries recorded were missed match injuries (see Table 2). There were more injuries classified as mild (41.6 [95% CI: 29.7 to 62.0] per 1000 match h) than moderate (RR: 4.0 [95% CI: 1.6 to 9.7]; *p* = 0.0010), slight (RR: 3.4 [95% CI: 1.5 to 7.9]; *p* = 0.0006), and minimum (RR: 2.7 [95% CI: 1.3 to 5.7]; *p* = 0.0090), and these were significant. Injuries recorded as mild had the highest injury probability (97.4%).

The lower limbs (105.6 [95% CI: 82.2 to 135.8] per 1000 match h) were recorded as having significantly more total injuries than the head/neck (RR: 2.3 [95% CI: 1.5 to 3.7]; *p* < 0.0001), chest/back/other (RR: 8.7 [95% CI: 4.0 to 18.9]; *p* < 0.0001), and upper limbs (RR: 20.3 [95% CI: 6.4 to 64.4]; *p* < 0.0001) (see Table 3). As a result, the lower limbs had the highest probability of an injury occurring for total (97.8%) and missed match (93.2%) injuries. The ankles (27.7 [95% CI: 17.0 to 45.2] per 1000 match h) were recorded as receiving significantly more MM injuries than the knees (RR: 5.3 [95% CI: 1.2 to 8.7]; *p* = 0.0164), hamstrings (RR: 5.3 [95% CI: 1.6 to 18.2]; *p* = 0.0029), and pelvis (RR: 8.0 [95% CI: 1.8 to 34.6]; *p* = 0.0010).

Sprains/strains were the most recorded total (86.6 [95% CI: 65.6 to 114.2] per 1000 match h) and MM (72.7 [95% CI: 53.7 to 98.4] per 1000 match h) injuries (see Table 3). The probability of a sprain/strain injury was 95.2% for total injuries and 91.6% for MM injuries. Fractures were recorded as having the highest mean injury burden (68.7 ± 70.4 days) of all the injury types.

## 4. Discussion

This prospective observational study documented match injury incidences in an amateur female soccer team over two consecutive domestic competition seasons. The findings can help inform targeted injury reduction programmes which address findings such as: (1) the total injury incidence was 145.5 (95% CI: 117.4 to 180.1) per 1000 match h; (2) the time-loss injury burden was 1796 days or a mean of 27.2 ± 38.0 days per injury; (3) the second quarter of match participation had the highest number of total and missed match injuries; (4) twisting (joint) had the highest total and MM injury rate, but overuse injuries resulted in higher total and MM mean days lost than any other match event; (5) sprains and strains were recorded as having the highest total and MM injury incidence; and (6) injuries to the lower limb body region resulted in the most days lost, with ankle injury being the most common total and missed match injury.

Interestingly the incidence of total (165.0 vs. 124.8 per 1000 match h) and MM (131.3 vs. 96.3 per 1000 match h) injuries decreased in the study second year. There was a change in coaches during the off season, and several players in the first year of the study left the team to play in other teams. Whilst these changes may have had an influence on the injury incidence, this would require further investigation into reasons why the incidence of match injuries decreased in the second competition year.

There was a total match injury incidence of 145.5 injuries per 1000 match h and a MM total injury incidence of 114.3 per 1000 match h. These findings are higher than those of previous studies reporting on female soccer players (9.1 [18], 16.3 [19], and 63.9 [9] injuries per 1000 match h) and highlight that females may have a higher injury risk than male soccer players (31.8 [20], 46.9 [9], and 44.7 [21] per 1000 match h). The total injury burden for all injuries was 3194.8 per 1000 match h or 3110.0 per 1000 match h for MM injuries, with a mean of 22.6 days lost per total injury and 27.2 days lost per MM injury. This finding is notably higher than those reported for male (455.7 per 1000 match h) and female (421.3 per 1000 match h) international participants [20] and Irish elite (640.3 per 1000 match h) [22] female football players. However, there is a paucity of injury burden data for female soccer players [7], and future studies should look to include this data as it would enable identification of patterns and areas for injury reduction programmes.

The finding that more missed match injuries occurred in the second half of matches is similar to some [23,24] but not all [9] studies reporting female football injuries. The differences between the current and other published studies may be in the injury definition utilised, with some, [18,19] but not all [9] studies utilising a MM or ‘time-loss’ injury definition. Although a consensus statement has been established [10] for the definition of an injury and data collection procedures in soccer, this is more focused on data capture at the professional level of participation by full-time medical staff [25]. Importantly, professional teams typically have medical support staff such as physiotherapists and sports medicine specialists and therefore have resources to manage injuries that do not result in match time loss; however, these services are not always available to amateur level teams [26]. Throughout the study, it was anecdotally noted that no other team in the competition had medical support for injured players. In some instances, the team medic (DK) assisted with injuries for both teams. This lack of medical support among other teams underscores the importance of having injury surveillance and assessment tools that can be used by non-medically trained team members to ensure the wellbeing of female players. The finding that the lower limbs had the highest injury incidence (105.6 per 1000 match h) is similar to other studies in female soccer [6,7,27]. However, the finding that ankle injury was the most commonly reported (43.3 per 1000 match h) lower limb injury is similar to some [22] but not all [6,27] previous studies reporting on women’s soccer. The most common injury type over the duration of the study was sprains and strains (86.6 per 1000 match h), and this is similar to previous studies [6,9,27]. Of concern was the finding that the second most common injury was concussion (20.8 per 1000 match h). This is notably higher than previous studies (0.1 [28] to 0.2 [6,29] per 1000 match h) but may be related to the increased awareness around concussion and the use of the CCP with any player suspected of having a meaningful knock or showing any signs of a concussive injury.

It should be noted that there were no reported injuries to or discomfort in the breast of any of the participants over the duration of this study. Research [30] has identified that breast protection is not commonly worn by female contact sports participants and that nearly half (47.9%) [31] of female athletes experience a breast injury, have some form of mastalgia, or experience exercise-induced breast pain during sports participation [32]. In addition, it has been identified that less than 10% of participants will report these injuries to their coach or medical professional. Injuries to the breast can have negative performance effects and may have some long-term consequences, such as fat necrosis, for the female participant later in life [31]. Future research in female sports should include the recording and reporting of these injuries.

The study findings were limited to a single amateur female club-based soccer team over two consecutive domestic competition seasons. The incidence of injuries may not be reflective of all amateur female club-based soccer teams, as this was a small sample of New Zealand players from a single club and should not be generalized to other cohorts. In addition, the coaching staff changed over the duration of the study, and the different coaching styles and player participation may have influenced the injury incidence over the duration of the study. Nevertheless, the findings of this study contribute to increasing the evidence base concerning female soccer players and can be used to support the development and implementation of injury surveillance methods, analysis, and reporting, as well as injury reduction programmes for recreational to amateur female soccer players.

## 5. Conclusions

This study is the first to report on New Zealand amateur domestic female soccer players. The total match (145.5 injuries per 1000 match h) and MM (114.3 per 1000 match h) injury incidences were higher than in previous studies reporting on female soccer players, highlighting that females may have a higher injury risk than male soccer players. The differences between the current study and the other studies may be attributed to the injury definition utilized, with some, but not all, studies utilising a MM or ‘time-loss’ injury definition. Ankle injury was the most reported (43.3 per 1000 match h) lower limb injury, whilst the most common injury type was sprains and strains (86.6 per 1000 match h) over the duration of the study. There were no reported injuries to or discomfort in the breast of any of the participants over the duration of this study. There is a paucity of injury burden data for female football, and future studies should include this data to enable identification of patterns and areas for injury reduction programmes.

## Figures and Tables

**Table 1:** Number of players, player ages, total and time-loss injuries, injury rates, injuries per match, injury burdens, and match minutes per injury in a New Zealand amateur female soccer domestic club-based team over two consecutive years for total and time-loss injuries. Data are reported as the number of injuries per 1000 match hours with 95% confidence intervals.

	Total Match Injuries			Time-Loss Injuries		
	2022	2023	Total	2022	2023	Total
Number of players, n	29	31 ^a^	49 ^b^	-	-	-
Player age, yr. mean ± SD	20.4 ^d^ ± 7.2	25.1 ^c^ ± 10.8	23.0 ± 9.8	-	-	-
Injuries observed, n	49	35	84	39	27	66
Injuries expected, n	43.2	40.8	84	33.9	32.1	66
Injury rates per 1000 match hours, n (95% CI)	165.0 (124.7–218.3)	124.8 (89.6–173.8)	145.5 (117.4–180.1)	131.3 (95.9–179.7)	96.3 (66.0–140.4)	114.3 (89.8–145.5)
Injury burden per 1000 match h, d (95% CI)	3764.3 (2845–4980.7)	2591.8 (1860.9–3609.8)	3194.8 (2579.7–3956.6)	3253.2 (2996.1–5000.1)	2534.8 (1738.3–3696.2)	3110.0 (2443.3–3958.5)
No. matches played, n	18	17	35	18	17	35
Exposure h, n	297.0	280.5	577.5	297.0	280.5	577.5
H per injury, m (95% CI)	6.1 (2.7–13.4)	8.0 (4.0–16.0)	6.9 (3.3–14.5)	7.6 (3.7–15.5)	10.4 (5.7–19.1)	8.8 (4.5–17.0)
Total no. injuries per match, m (95% CI)	2.7 (0.8–8.9)	2.1 (0.5–8.1)	2.4 (0.7–8.5)	2.2 (0.6–8.2)	1.6 (0.3–7.5)	1.9 (0.5–7.9)
Player appearances per injury, m (95% CI)	4.0 (1.5–10.7)	5.3 (2.3–12.5)	4.6 (1.8–11.4)	5.1 (2.1–12.1)	6.9 (3.3–14.6)	5.8 (2.6–13.1)
Match minutes played per injury, m (95% CI)	33.1 (23.5–46.5)	43.7 (32.5–58.8)	37.5 (27.2–51.6)	41.5 (30.6–56.3)	56.7 (43.7–73.5)	47.7 (35.9–63.4)
Injury probability, %	99.9	98.7	100.0	99.6	96.6	100.0

^a^ = includes 11 players who were enrolled in 2022; ^b^ = total players enrolled over 2 yrs.; n = number; d = days; m = median; CI = confidence interval; SD = standard deviation; significant difference (*p* < 0.05) from (^c^) 2022; (^d^) 2023.

**Table 2:** Player group, match period, injury cause, and injury severity for injuries that occurred in a New Zealand amateur female domestic club-based football team for total injuries and time-loss injuries over two consecutive years by the number of injuries recorded, rates per 1000 match hours with 95% confidence intervals, injury probability, number of days lost, and mean number of days lost with standard deviation.

	Total Injuries					Time Loss Injuries				
	Number of Injuries			Injury Burden		Number of Injuries			Injury Burden	
	n =	Injury Incidence	Inj Prob	Days Lost			Injury Incidence	Inj Prob	Days Lost	
		Rate (95% CI)	%	Total	Mean ± SD	n =	Rate (95% CI)	%	Total	Mean ± SD
Total	84	145.5 (117.4–180.1)	99.6%	1845	22.6 ± 36.8	66	110.8 (86.7–141.6)	98.5%	1796	27.2 ± 38.00
Player position										
Goalie, n = 1	5	95.2 (39.6–228.8)	71.1%	226 ^bcd^	45.2 ± 59.5	4	38.1 (9.5–152.3)	74.7%	222 ^bcd^	55.5 ± 63.4
Defender, n = 4	32	152.4 (107.8–215.5)	84.1%	479 ^acd^	15.0 ± 12.4	24	114.3 (76.6–170.5)	75.4%	458 ^acd^	19.1 ± 11.6
Midfielder, n = 4	26	123.8 (84.3–181.8)	77.8%	578 ^abd^	22.2 ± 41.3	22	104.8 (69.0–159.1)	72.9%	568 ^abd^	25.8 ± 44.1
Attacker, n = 2	21	200.0 (130.4–306.7)	71.7%	562 ^abc^	26.8 ± 42.3	16	152.4 (93.4–248.7)	66.0%	548 ^abc^	34.3 ± 46.2
Match duration										
1st quarter	7 ^fgh^	48.5 (23.1–101.7)	66.3%	104 ^fgh^	14.9 ± 13.5	5 ^fgh^	34.6 (14.4–83.2)	71.1%	96 ^fgh^	19.2 ± 13.8
2nd quarter	35 ^eh^	242.4 (174.1–337.6)	86.8%	708 ^eg^	20.2 ± 25.9	25 ^e^	173.2 (117.0–256.3)	76.6%	681 ^e^	27.2 ± 27.7
3rd quarter	22 ^e^	152.4 (100.3–231.4)	72.9%	292 ^efh^	13.3 ± 9.1	19 ^e^	131.6 (83.9–206.3)	69.3%	284 ^e^	14.9 ± 8.7
4th quarter	20 ^ef^	138.5 (89.4–214.7)	70.8%	741 ^eg^	37.1 ± 60.7	17 ^e^	117.7 (73.2–189.4)	67.0%	735 ^e^	43.2 ± 64.0
1st half	42	145.5 (107.5–196.8)	91.6%	812 ^j^	19.3 ± 24.2	30	103.9 (72.6–148.6)	82.2%	777 ^j^	25.9 ± 25.9
2nd half	42	145.5 (107.5–196.8)	91.6%	1033 ^i^	24.6 ± 43.5	36	124.7 (89.9–172.8)	87.6%	1019 ^i^	28.3 ± 46.0
Injury cause										
Twist	27 ^mnopqrs^	46.8 (32.1–68.2)	78.9%	495 ^lnopqrs^	18.3 ± 41.2	18 ^p^	31.2 (19.6–49.5)	68.1%	419 ^lnoprs^	29.9 ± 55.5
Hyperextension	20 ^opqrs^	32.9 (21.0–51.6)	70.5%	262 ^kmnpqrs^	13.1 ± 7.1	17	29.4 (18.3–47.4)	67.0%	219 ^kmprs^	15.6 ± 6.4
Surface	11 ^kqrs^	19.0 (10.5–34.4)	63.2%	453 ^lnopqrs^	41.2 ± 55.6	9	15.6 (8.1–30.0)	63.9%	449 ^lnoprs^	49 + 0.9 ± 58.3
Player	10 ^kqrs^	17.3 (9.3–32.2)	63.4%	218 ^klmpqrs^	21.8 ± 9.1	10 ^re^	17.3 (9.3–32.2)	63.4%	218 ^kmoprs^	21.8 ± 9.1
Ball	9 ^klqrs^	15.6 (8.1–30.0)	63.9%	233 ^kmpqrs^	25.9 ± 12.3	9	15.6 (8.1–30.0)	63.9%	233 ^kmnprs^	25.9 ± 12.3
Overuse	3 ^klrs^	5.2 (1.7–16.1)	79.2%	168 ^klmnoqrs^	56.0 ± 81.6	2 ^k^	3.5 (0.9–13.8)	84.7%	164 ^kmnrs^	82.0 ± 96.2
Temperature	2 ^klmno^	3.5 (0.9–13.8)		6 ^klmnop^	3.0 ± 0.0	0	0.0 -	-	0	0 -
Other	1 ^klmnop^	1.7 (0.2–12.3)	91.7%	14 ^klmnop^	14.0 -	0	0.0 -	-	0	0 -
Fall	1 ^klmnop^	1.7 (0.2–12.3)	91.7%	10 ^klmnop^	10 -	1 ^n^	1.7 (0.2–12.3)	91.7%	10 ^klmnop^	10.0 -
Injury severity										
Slight	8 ^vx^	12.1 (5.8–25.4)	64.9%	13 ^uvwx^	1.9 ± 0.4	-	-	-	-	-
Minimal	10 ^vx^	15.6 (8.1–30.0)	63.4%	30 ^tvwx^	3.3 ± 0.58	-	-	-	-	-
Mild	40 ^tuw^	41.6 (27.9–62.0)	90.7%	462 ^tuwx^	11.6 ± 2.4	40 ^w^	41.6 (27.9–62.0)	90.7%	462 ^wx^	11.6 ± 2.4
Moderate	6 ^vx^	10.4 (4.7–23.1)	68.4%	144 ^tuvx^	24.0 ± 2.6	6 ^vx^	10.4 (4.7–23.1)	68.4%	144 ^vx^	24.0 ± 2.6
Severe	20 ^tuw^	34.6 (22.3–53.7)	70.5%	1190 ^tuvw^	29.5 ± 57.5	20 ^w^	34.6 (22.3–53.7)	70.5%	1190 ^vw^	29.5 ± 57.5
Transient	18 ^z^	27.7 (17.0–45.2)	68.1%	43 ^z^	2.7 ± 0.9	-	-		-	-
Missed match	66 ^y^	86.6 (65.6–114.2)	98.5%	1796 ^y^	27.2 ± 38.0	66	86.6 (65.6–114.2)	98.5%	1796	27.2 ± 38.0

n = number; CI = confidence interval; SD = standard deviation; Inj Prob = injury probability; significant difference (*p* < 0.05) from (^a^) = goalie; (^b^) = defender; (^c^) = midfielder; (^d^) = attacker; (^e^) = 1st quarter; (^f^) = 2nd quarter; (^g^) = 3rd quarter; (^h^) = 4th quarter; (^i^) = 1st half; (^j^) = 2nd half; (^k^) = twist; (^l^) = hyperextension; (^m^) = surface; (^n^) = player; (^o^) = ball; (^p^) = overuse; (^q^) = temperature; (^r^) = other; (^s^) = fall; (^t^) = slight; (^u^) = minimal; (^v^) = mild; (^w^) = moderate; (^x^) = severe; (^y^) = transient; (^z^) = missed match.

**Table 3:** Injury sites and injury types for injuries that occurred in a New Zealand amateur female domestic club-based football team for total injuries and time-loss injuries over two consecutive years by the number of injuries recorded, rates per 1000 match hours with 95% confidence intervals, injury probability, number of days lost, and mean number of days lost with standard deviation.

	Total Injuries					Missed-Match				
	Number of Injuries		Inj Prob	Total Injury Burden		Number of Injuries		Inj Prob	Total Injury Burden	
	Injury Incidence			Days Lost		Injury Incidence			Days Lost	
	n =	Rate (95% CI)	%	n =	Mean ± SD	n =	Rate (95% CI)	%	n =	Mean ± SD
**Injury site**										
**Head/neck**										
**Total**	**26 ^bcd^**	**45.0 (30.7–66.1)**	**77.8%**	**762 ^bcd^**	**29.3 ± 7.6**	**26 ^bcd^**	**45.0 (30.7–66.1)**	**77.8%**	**762 ^bcd^**	**29.3 ± 7.6**
Head	12 ^fhiknoqrst^	20.8 (11.8–36.6)	63.4%	372 ^fhijklmnopqrst^	31.0 ± 4.9	12 ^fhikors^	20.8 (11.8–36.6)	63.4%	372 ^fhijklmnoprs^	31.0 ± 4.9
Face	1 ^egjlmnp^	1.7 (0.2–12.3)	91.7%	9 ^egijklmnoprs^	9.0 -	1 ^egjmn^	1.7 (0.2–12.3)	91.7%	9 ^egijklmnqr^	9.0 -
Neck	13 ^fhikoqrst^	22.5 (13.1–38.8)	63.8%	381 ^fhijklmnopqrst^	29.3 ± 7.7	13 ^fhikors^	22.5 (13.1–38.8)	63.8%	381 ^fhijklmnoprs^	29.3 ± 7.7
**Upper limb**										
**Total**	**3 ^ac^**	**5.2 (1.7–16.1)**	**79.2%**	**38 ^acd^**	**12.7 ± 2.3**	**3 ^ac^**	**5.2 (1.7–16.1)**	**79.2%**	**38 ^acd^**	**12.7 ± 2.3**
Wrist	1 ^egjlmnp^	1.7 (0.2–12.3)	91.7%	14 ^egjklmnpqrs^	14 -	1 ^egjmn^	1.7 (0.2–12.3)	91.7%	14 ^egjklmnprs^	14 -
Finger	2 ^egn^	3.5 (0.9–13.8)	84.8%	24 ^efgjklmnpqst^	12.0 ± 2.8	2 ^egn^	3.5 (0.9–13.8)	84.8%	24 ^efgjklmnps^	12.0 ± 2.8
**Lower limb**										
**Total**	**61 ^abd^**	**105.6 (82.2–135.8)**	**97.8%**	**1227 ^abd^**	**20.1 ± 37.1**	**45 ^abd^**	**77.9 (58.2–104.4)**	**93.2%**	**1184 ^abd^**	**26.3 ± 41.6**
Quadriceps	7 ^fno^	12.1 (5.8–25.4)	66.3%	116 ^efghiklmnopqrst^	16.6 ± 7.0	7 ^fho^	12.1 (5.8–25.4)	66.3%	116 ^efghiklmnoprs^	16.6 ± 7.0
Hamstring	3 ^eghn^	5.2 (1.7–16.1)	79.2%	58 ^efghijlmnopqrst^	19.3 ± 6.4	3 ^egn^	5.2 (1.7–16.1)	79.2%	58 ^efghijl^	19.3 ± 6.4
Knee	7 ^fno^	12.1 (5.8–25.4)	66.3%	270 ^efghijkmnopqrst^	38.6 ± 80.1	5 ^n^	8.7 (3.6–20.8)	71.1%	264 ^efghijkmnoprs^	52.8 ± 93.5
Lower leg	8 ^fhno^	13.9 (6.9–27.7)	64.9%	82 ^efghijklnoqrst^	10.3 ± 4.2	7 ^fho^	12.1 (5.8–25.4)	66.3%	80 ^efghijlnoprs^	11.4 ± 2.7
Ankle	25 ^efhijklmopqrst^	43.3 (29.3–64.1)	76.6%	596 ^efghijklmopqrst^	23.8 ± 39.9	16 ^fhikloprs^	27.7 (17.0–45.2)	66.0%	545 ^efghijlmoprs^	38.9 ± 48.7
Achilles	1 ^egjlmnp^	1.7 (0.2–12.3)	91.7%	14 ^egjklmnpqrs^	14 -	1 ^egjmn^	1.7 (0.2–12.3)	91.7%	14 ^egjlmnprs^	14 -
Foot	8 ^fhno^	13.9 (6.9–27.7)	64.9%	87 ^efghijklnoqrst^	10.9 ± 7.9	6 ^n^	10.4 (4.7–23.1)	68.4%	81 ^eghijlnors^	13.5 ± 7.4
Toe	2 ^egn^	3.5 (0.9–13.8)	84.8%	4 ^efghijklmnoprs^	2.0 ± 0.0	0	0.0 -	-	0	0 -±
**Chest/back/other**										
**Total**	**7 ^ac^**	**12.1 (5.8–25.4)**	**66.3%**	**199 ^abc^**	**28.4 ± 53.7**	**5 ^ac^**	**8.7 (3.6–20.8)**	**71.1%**	**193 ^abc^**	**38.6 ± 62.3**
Lower back	3 ^egn^	5.2 (1.7–16.1)	79.2%	34 ^efghjklmnoprst^	11.3 ± 1.2	3 ^egn^	5.2 (1.7–16.1)	79.2%	34 ^efgjlmnops^	11.3 ± 1.2
Pelvis	2 ^egn^	3.5 (0.9–13.8)	84.8%	159 ^efghijklmnopqt^	79.5 ± 99.7	2 ^egn^	3.5 (0.9–13.8)	84.8%	159 ^efgijlmnopr^	79.5 ± 99.7
Other	2 ^egn^	3.5 (0.9–13.8)	84.8%	6 ^egijklmnpqs^	3.0 ± 0.0	0	0.0 -	-	0	0 -
**Total**	**97**	**168 (137.7–204.9)**	99.9%	**2226**	**22.9 ± 32.8**	**79**	**136.8 (109.7–170.5)**	99.5%	**2177**	**27.6 ± 34.8**
**Injury type**										
Strains/sprains	50 ^vwxyz†⁋^	86.6 (65.6–114.2)	95.2%	1165 ^vwxzy†⁋^	23.3 ± 40.2	42 ^vwxyz^	72.7 (53.7–98.4)	91.6%	1132 ^vwxyz^	29.0 ± 44.3
Concussion	12 ^uz†⁋^	20.8 (11.8–36.6)	63.4%	372 ^uwxyz†⁋^	31.0 4.9±	12 ^uxyz^	20.8 (11.8–36.6)	63.4%	372 ^uwxyz^	31.0 ± 4.9
Contusion	11 ^uz†⁋^	19.0 (10.5–34.4)	63.2%	90 ^uvxyz†⁋^	8.2 ± 4.5	8 ^u^	13.9 (6.9–27.7)	64.9%	84 ^uvxyz^	10.5 ± 2.5
Cramp	6 ^u^	10.4 (4.7–23.1)	68.4%	37 ^uvwz†⁋^	6.2 ± 4.1	3 ^uv^	5.2 (1.7–16.1)	79.2%	29 ^uvwyz^	9.7 ± 2.1
Abrasion	5 ^u^	8.7 (3.6–20.8)	71.1%	54 ^uvwz†⁋^	10.8 ± 10.8	3 ^uv^	5.2 (1.7–16.1)	79.2%	50 ^uvwxz^	16.7 ± 10.3
Fracture	3 ^uvw^	5.2 (1.7–16.1)	79.2%	206 ^uvwxy†⁋^	68.7 ± 70.4	3 ^uv^	5.2 (1.7–16.1)	79.2%	206 ^uvwxy^	68.7 ± 70.4
Blister	2 ^uvw^	3.5 (0.9–13.8)	84.8%	4 ^uvwxyz^	2.0 ± 0.0	0	0.0 -	-	0	0 -
Other	2 ^uvw^	3.5 (0.9–13.8)	84.8%	6 ^uvwxyz^	3.0 ± 0.0	0	0.0 -	-	0	0 -

CI = confidence intervals; SD = standard deviation; Inj Prob = injury probability; significant difference (*p* < 0.05) from (^a^) = head/neck; (^b^) = upper limb; (^c^) = lower limb; (^d^) = chest/back/other; (^e^) = head; (^f^) = face; (^g^) = neck; (^h^) = wrist; (^i^) = finger; (^j^) = quadriceps; (^k^) = hamstring; (^l^) = knee; (^m^) = lower leg; (^n^) = ankle; (^o^) = achilles; (^p^) = foot; (^q^) = toe; (^r^) = lower back; (^s^) = pelvis; (^t^) = other; (^u^) = strains/sprains; (^v^) = concussion; (^w^) = contusion; (^x^) = cramp; (^y^) = abrasion; (^z^) = fracture; (^†^) = blister; (^⁋^) = other.

## Data Availability

The statistical analysis for the study was undertaken using the Statistical Package for Social Sciences for Windows (SPSS; V25.0.0). Data are available from the lead author on request.
